# *oxi-1* and *fshr-1* are required for neuromuscular signaling under normal and oxidative stress conditions in C. elegans

**DOI:** 10.17912/pfyw-ft85

**Published:** 2018-08-28

**Authors:** Barry Wei, Jennifer R. Kowalski

**Affiliations:** 1 Butler University, Department of Biological Sciences, Indianapolis, IN 46208

**Figure 1. oxi-1 and fshr-1 loss-of-function mutants exhibit reduced body bending rates compared to wild type N2 worms under both normal and oxidative stress conditions.**
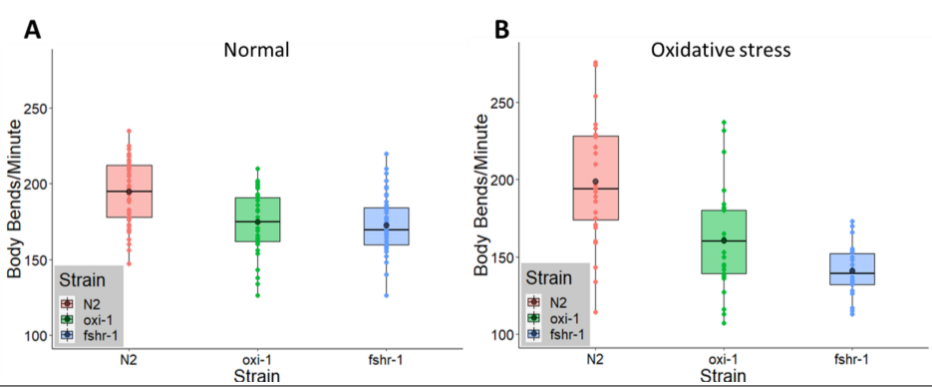
**(A)** *oxi-1(ok1217)* and *fshr-1(ok778)* mutants showed 11.0% (*p* = < 2.1167E-050.0001) and 11.2 % (*p* = 3.8259E-06< 0.00001) reductions in body bends, respectively, compared to N2 animals when tested in the absence of prior oxidative stress. Worms were young adult aged and grown at 20 º C (n = 33 N2, 33 *oxi-1*, and 44 *fshr-1*). **(B)** *oxi-1(ok1217)* and *fshr-1(ok778)* mutants exposed to oxidative stress both had further reduced body bending rates compared to N2 worms with differences of 19.2% (*p* < 1.77E-040.001) and 29.2% (*p* = < 2.1771E-080.0000001), respectively. Worms were exposed to 5 mM paraquat for 48 hours beginning at the L3/L4 stage prior to being assayed for body bends as young adults (n = 28 N2, 29 *oxi-1*, and 22 *fshr-1*).

## Description

Reactive oxygen species (ROS) contribute to neuronal degeneration by readily reacting with cellular components, consequently breaking down cellular integrity. Excess ROS often leads to oxidative stress, which results from destabilization of the organism’s ability to control the balance between antioxidants and free radicals (Chandra *et al.* 2015). The ubiquitin-proteasome system helps to regulate oxidative stress and overall damage to cellular components by forming chains of ubiquitin polypeptides on cellular proteins; these chains then serve as a signal to break down the attached protein (Hershko *et al.* 1983). Mutation of *UBE3B*, an E3 ubiquitin ligase, has been found to lead to Blepharophimosis-Ptosis-Intellectual-Disability Syndrome (BPID) in human infants, indicating potential involvement of *UBE3B* in the regulation of neuronal signaling in the brain (Basel-Vanagaite *et al.* 2012). The UBE3B protein was also shown to be involved in mitochondrial function and oxidative stress responses in mammalian cells (Braganza *et al.* 2017). In *C. elegans*, the *oxi-1* gene encodes an ubiquitin ligase homologous to UBE3B (58% amino acid similarity); expression of *C. elegans oxi-1* is induced by oxidative stress and is required for proteasomal responses to this stress (Basel-Vanagaite *et al.* 2012). However, despite the link to neurodevelopmental disorders including BPID, specific roles for *UBE3B* or *oxi-1* in neuronal biology and synaptic function, with or without oxidative stress, have not been explored (Basel-Vanagaite *et al*. 2012). A second *C. elegans* gene *fshr-1*, which encodes a G protein-coupled receptor homologous to a family of mammalian glycopeptide hormone receptors (Cho *et al,* 2007), is also involved in both oxidative stress responses and neuronal signaling. Specifically, *fshr-1* regulates expression of *gcs-1*, an oxidative-stress response gene (Miller *et al*. 2015) and was identified in an RNAi interference screen as a gene required for proper structure and function of neuromuscular synapses (Sieburth *et al.* 2005). Despite data suggesting roles for *oxi-1 UBE3B*, and *fshr-1* in neuronal signaling, which is susceptible to oxidative damage, neither gene has been investigated with regards to neuronal signaling in the presence of oxidative stress. Here, we tested the requirements of both *oxi-1* and *fshr-1* for their effects on neuromuscular signaling under both normal and oxidative stress conditions in *C. elegans.*

Neuronal signaling activity can be measured at the neuromuscular junction in *C. elegans*, a model synapse, by observing the motility of individual worms in liquid medium in a body bending assay (Nawa and Matsuoka 2012). Under normal conditions, *C. elegans* mutants lacking expression of either *oxi-1* or *fshr-1* had reduced motility compared to wild type animals by 11.0% (*p* < 0.0001= 2.1167E-05) and 11.2% (*p* < 0.00001= 3.8259E-06), respectively (**Figure 1A**). Following exposure to oxidative stress conditions (5 mM paraquat) for 48 hours, the mean number of body bends for wild type animals remained constant (normal: 194 body bends/minute, oxidative stress: 199 body bends/minute). However, the motility of *oxi-1* mutants compared to wild type N2 worms under oxidative stress conditions in the body bending assay decreased from 173 to 160.5 body bends/minute, a 19.2% reduction compared to the wild type strain (*p* < 0.001= 1.77E-04) (**Figure 1B**). An even larger decrease in body bends, from 172.5 to 140.8 body bends/minute, a decrease of 29.2% (*p* = < 0.00000012.1771E-08) was observed for *fshr-1* mutants compared to wild type animals under oxidative stress conditions (**Figure 1B**). Successful induction of oxidative stress was determined for each body bending assay by assessing whether a separate strain of worms (*ldIs3*) carrying the oxidative stress responsive reporter *gcs-1p::GFP* exposed to paraquat alongside *oxi-1*, N2, and *fshr-1* expressed green fluorescence when observed under a fluorescence stereomicroscope.

## Methods

During the body bending assay, young adult worms of each strain were individually isolated on unspotted NGM agar plates, and each worm was allowed to move around for 1-2 minutes to dislodge any chunks of bacteria from its body. Then, the worm was placed into its own well containing 100 µL of M9 salt buffer and allowed one minute for acclimation. Afterwards, observation of the worm’s number of body bends over the course of one minute was recorded. Note that a single body bend is defined by the movement of the worm’s head and tail from its initial position to one side and then back (Nawa and Matsuoka 2012).

Using the recorded body bending rates of each strain under both normal and oxidative stress conditions, normality of the data distribution for each strain was first checked using a normal probability plot and/or applying the central limit theorem when sample size was greater than or equal to thirty. Once all datasets were confirmed to be normally distributed, the mean and standard deviation were computed in Microsoft Excel. An F test for equality of variances for N2 compared to each mutant strain under each condition was performed. Statistical significance of the differences in mean body bends between N2 and each mutant strain was determined using two-tailed Student’s *t*-tests assuming equal variance for all pairs except for N2 vs. *oxi-1* under oxidative stress, for which the *t* test was done assuming unequal variance based on the results of the F test (*p* = < 0.00017001). For graphical data representation, a boxplot and dot plot were layered on top of each other, including a black dot to represent the distribution mean for each strain, using the ggplot2 function in R.

## Reagents

***Strains:*** N2*,* RB1176 *oxi-1(ok1217)*, RB911 *fshr-1(ok778)*, LD1171 *lIdIs3* [*gcs-1p::GFP + rol-6*]. All strains are available at the CGC.

***Materials:*** M9 buffer, Paraquat (methyl viologen dichloride hydrate, Sigma-Aldrich #856177), NGM agar, OP50 *Escherichia coli*, 35 mm petri dishes, 96 micro-well plate

***Worm Growth Conditions*:** All strains were grown at constant 20 º C; for assays, worms were plated at the L3/L4 stage onto 35mm NGM agar plates +/- a final paraquat concentration of 5 mM, and grown until the young adult stage at which time body bending assays were performed.
